# The Effectiveness of Commercial Vaccination against *Lawsonia intracellularis* in Mitigating the Reduction in ADWG, the Increased Mortality and Fecal Shedding of the Vaccinated Pigs: A Systematic Review and Meta-Analysis

**DOI:** 10.3390/vetsci9100536

**Published:** 2022-09-28

**Authors:** Gayeon Won, Na-Kyoung Chi, Yebin Park

**Affiliations:** College of Veterinary Medicine, Jeonbuk National University, Iksan Campus, Gobong-ro 79, Iksan 54596, Korea

**Keywords:** *Lawsonia intracellularis*, proliferative enteropathy, vaccine, intervention, meta-analysis, systematic review

## Abstract

**Simple Summary:**

Porcine proliferative enteropathy (PPE), caused by *Lawsonia intracellularis,* is prevalent globally and produces a great economic impact on affected countries. However, its pathogenic mechanism has not been completely elucidated as the infectious agent is an anaerobic obligate intracellular bacterium, which has resulted in considerable difficulty for controlling the disease. Two commercial vaccines for controlling PPE are currently available, and a systematic review and meta-analysis were performed to assess the pooled effect of the vaccines to provide updated quantitative evidences to the stakeholders. The efficacy of the vaccines was assessed using three outcomes: average daily weight gain (ADWG), mortality, and fecal shedding. The results indicated that the vaccinated pigs showed significantly reduced risk in mortality and fecal-shedding compared to the control pigs. The significant increase in ADWG was also observed in the immunized pigs compared to the unvaccinated controls. Considering the current situation that new alternatives for control of PPE is not identified, these evidence-based findings will help improve decision-making on practical use of the vaccines to prevent PPE. Furthermore, the results will also provide updated information to the researchers while experimental candidate vaccines against PPE are being developed.

**Abstract:**

In this study, a systematic review and meta-analysis was conducted to assess the efficacy of commercial vaccines against PPE in accordance with the Preferred Reporting Items for Systematic Reviews and Meta-Analyses (PRISMA) checklist. Of the 373 articles reviewed, 16 fulfilled the pre-specified inclusion criteria. Three independent reviewers extracted the data, and vaccine effectiveness was assessed using the outcomes of interest. The majority of studies had a low or unclear risk of bias as assessed using the ARRIVE guidelines. The results of the meta-analysis indicated that the vaccination resulted in statistically significant reductions in bacterial fecal shedding (odds ratio, OR = 0.122, 95% confidence interval, CI 0.054–0.278) and mortality rate (risk ratio, RR = 0.199; 95% CI, 0.066–0.605). Furthermore, ADWG was significantly increased in the vaccinated pigs compared to the unvaccinated controls (standardized mean difference (SMD) = 0.606, 95% CI 0.243–0.969). In the subgroup analysis, the production phase and study type significantly influenced the effect size (*p* < 0.1). The Egger’s regression test showed no evidence of publication bias (*p* > 0.1). The effectiveness of commercially available vaccines against PPE-related weight loss, fecal shedding, and mortality suggests that the vaccines may help control PPE on affected swine farms.

## 1. Introduction

Porcine proliferative enteropathy (PPE) is a highly endemic bacterial disease that poses a considerable economic burden, and it has a high herd-level prevalence ranging from 48% to 100% at swine production sites globally [1]. *Lawsonia intracellularis* (*L. intracellularis*) is an obligate intracellular microorganism that causes PPE, which is characterized by hyperplasia of crypt enterocytes in the ileum and colon, leading to several clinical manifestations. PPE has two major clinical forms: acute proliferative hemorrhagic enteropathy (PHE) and chronic porcine intestinal adenomatosis (PIA). The acute form causes hemorrhagic diarrhea and results in sudden death, mainly in young adult pigs at 4–12 months of age. The chronic form is characterized by the clinical manifestation of diarrhea or subclinical infection, leading to weight and productivity losses, particularly in pigs aged 6–20 weeks [2]. Control and treatment of PPE has been challenging as an accurate defense mechanism against *L. intracellularis* infection has not been fully elucidated. Owing to the fastidious characteristics of *L. intracellularis*, the obligate anaerobic bacteria are extremely difficult to culture in vitro. 

Currently, two different types of *L. intracellularis* vaccines are commercially available: live attenuated (Enterisol^®^ Ileitis, Boehringer Ingelheim B.V., Alkmaar, The Netherlands) [3] and an inactivated bacteria-based vaccine (Porcilis^®^ Lawsonia, Merck Animal Health, Madison, NJ, USA) [4]. A recently published literature review reported that live and inactivated vaccines licensed in 2001 and 2015, respectively, resulted in successful induction of humoral and cell-mediated immune responses but failed to show whether sterile immunity was conferred in the affected animals [5]. Several studies have reported the effectiveness of commercial vaccines in preventing PPE [3,6,7]; however, in order to conduct an integrated evaluation of the vaccine effectiveness (VE), a correction for multiple comparisons is required due to substantial variations among experimental design factors such as study type, sample size, and challenge type. In this regard, the introduction of a comprehensive approach combining currently available data may guide decisions regarding relevant vaccine research in the future.

In this study, we conducted a systematic review and meta-analysis of eligible studies that determined the efficacy of commercial vaccines against *L. intracellularis* to provide consistent information on their effectiveness. Meta-analyses are quantitative methods for increasing statistical power by combining the numerical results of multiple well-designed studies, thereby allowing the detection of a more precise estimate of the effectiveness of intervention strategies than in any individual study [8]. The objective of this investigation was to conduct a systematic meta-analysis of published studies for the vaccine efficacy in controlling shedding of *L. intracellularis,* and effects of *L. intracellularis* infection on ADWG and fatality. Eligible studies describing commercial swine vaccines against *L. intracellularis* published in peer-reviewed journals were identified and evaluated using a structured review process. A meta-analysis was used to compute the pooled effect size and to predict the effectiveness of the vaccines for the prevention of a decrease in the average daily weight, bacterial fecal shedding, and mortality rate. This is the first systematic review and meta-analysis of swine vaccines against *L. intracellularis*, facilitating a better understanding of current PPE prevention strategies. 

## 2. Methods

The review process and reporting of outcomes adhered to the Preferred Reporting Items for Systematic Reviews and Meta-Analyses (PRISMA) statement [9].

### 2.1. Search Strategy and Selection Criteria

The search terms satisfied the population, intervention, comparator, and outcome (PICO) format. The population of interest was swine in any production phase worldwide. The intervention arms were commercially available live-attenuated or inactivated vaccines that protect against PPE. The comparator included negative control pigs (sham-vaccinated or unvaccinated). For outcomes, at least one of the following measurements was assessed: mortality, fecal shedding rate, or average daily weight gain (ADWG).

Four key indexing and abstracting services (PubMed, AGRICOLA, CAB abstracts, and Google Scholar) for veterinary science were searched in March 2021 to identify primary scientific literature. Gray literature was searched using Google Scholar, and the first 100 abstracts sorted by relevance were evaluated. For all four databases, the following keywords were used to select relevant studies: (lawsonia* OR “proliferative enteropathy”) AND (immuni* OR vaccin* OR interve* OR Enterisol OR treatment OR efficacy OR effect OR protect OR shed) AND (vaccin* OR immunis* OR immuniz* OR “mitigat* OR interve* OR control) AND (swine OR pig or pigs OR piglet OR piglets OR gilt OR gilts OR sow OR sows OR hog OR hogs OR weaner OR feeders OR finisher OR finishers OR porcine OR pork). The asterisk was applied to extend the search for related words; for example, interve* searches for intervention and interventions. The search results were imported into Endnote bibliographic management software version X9.3 (Thomson Reuters, Carlsbad, CA, USA), and duplicate literature was identified.

### 2.2. Study Selection Process

After eliminating duplicates, the relevant articles were screened using their abstracts, titles, and full texts from the electronic database, if needed. The screened studies were considered eligible if they met the following inclusion criteria: (1) peer-reviewed original articles; (2) primary research; (3) commercial vaccines were used; (4) vaccine effectiveness in a trial under natural exposure field trials or artificial challenge studies was evaluated; and (5) a control group of pigs (i.e., sham-vaccinated or unvaccinated) was included. There were no language restrictions for inclusion. Review papers, conference proceedings, and case reports that provided insufficient information were excluded. Reporting of experimental vaccines was also deemed ineligible.

### 2.3. Data Extraction

Two reviewers independently extracted the following data in a predetermined form: (1) basic information, including title, authors, publication year, study, or trial setting (i.e., research vs. commercial farm); (2) characteristics of participants (i.e., age at vaccination, production phase, and sample size of vaccinated and controlled pigs); (3) characteristics of intervention arms (including vaccine type, dose, frequency, or route of administration); and (4) eligible outcomes including ADWG, fecal shedding, and mortality rate. Proxy outcomes for the potential protective effects of immunization, such as an increase in humoral or cell-mediated immunogenicity, were not included in the analysis. Histopathological outcomes such as lesion scores estimating the proportion of crypt hyperplasia were also excluded because of the substantial risk of observer bias. The terms “study” or “trial” were defined by the selected studies, and subgroup data were collected within the study. Studies providing ADWG as an outcome were classified according to the production phase (i.e., nursery and growing-finishing phases) when the weight was measured. For the fecal shedding rate outcome, studies employing polymerase chain reaction (PCR) to determine the presence of LI genomic DNA were included in the meta-analysis for consistency only. The fecal shedding rate measured using the cell culture protocol was excluded owing to the characteristics of obligate anaerobic bacteria LI, which are extremely difficult to cultivate in vitro. Furthermore, since immunity against LI was generally achieved 7–10 days after primary vaccination, the number of bacterial DNA-positive fecal samples observed more than 7 days following the vaccination was counted to examine the effect of the intervention.

### 2.4. Quality Assessment

ARRIVE 2.0 (Animal Research: Reporting in Vivo Experiments) guidelines [10] were used to assess the potential risk of bias of the included articles. Three reviewers (G.W., N.-K.C. and Y.P.) independently evaluated the study quality based on the following Cochrane risk-of-bias domains: random sequence generation, allocation concealment, blinding of participants and personnel, blinding of outcome assessment, incomplete outcome data, selective reporting, and other sources of bias. The risk of bias based on the outcome of each study was categorized as “low” “high”, or “unclear”. Any discrepancies were resolved by consensus of the reviewers.

### 2.5. Outcome Measures

For the outcome measured on a continuous scale (i.e., ADWG), the standardized mean difference (SMD) and 95% confidence interval (CI) were computed using means and measures of variability (i.e., standard deviation, SD). For missing SDs, the values were calculated using the standard error of the mean (SEM) and the sample size or imputed using the overall effect estimate according to a published method [11]. To remove the potential bias introduced by the method, we conducted a sensitivity analysis for which the overall estimated effect size was compared with that of the group, excluding studies that did not report SDs. When the outcome data were shown only in a graph with numeric labels, they were extracted from the graphs as necessary. For dichotomous measures (i.e., bacterial fecal shedding or mortality), the risk ratio (RR) or odds ratios (OR) for vaccinated animals and controls were employed as the effect size metric. The vaccination was considered effective when the pooled estimate of RR or OR and the 95% confidence interval were below the null value (RR or OR = 1).

### 2.6. Data Analysis

We performed a meta-analysis to assess the effectiveness of a commercial swine vaccine in the prevention of PPE, using a fixed-effects or random-effects model. The degree of heterogeneity or variability of each outcome among the trials was evaluated using Cochran’s Q and Higgins’ inconsistency (*I*^2^) statistics [12]. A value of *I*^2^ higher than 75% indicated a potential source of heterogeneity. For the trials containing a fecal-shedding outcome (OR), the meta-analysis was carried out by adapting fixed-effect models because of the low number of eligible studies. The random-effects models, which incorporated a weighted inter-study variance (*τ*^2^) in the meta-analysis, were applied to pool the mortality risk ratios and SMD measures for ADWG. Subsequently, a subgroup analysis was performed; a mixed-effect model was used to explore the variables (i.e., moderator) that were significantly related to heterogeneity among the studies. The mixed-effects model assumes that sampling errors are randomly generated, but other sources of variation originate from random or systematic errors, such as the presence of moderators [13]. A *p*-value for the difference among subgroups of <0.10 was considered statistically significant. Publication bias was evaluated using Egger’s regression test and was graphically represented by funnel plots. Egger’s regression test (*p*-value < 0.05) was considered statistically significant. If a significant bias was reported, the trim-and-fill method of Duvall and Tweedie was used to determine the impact of the bias on the estimated effect size. We completed all analyses using Comprehensive Meta-Analysis, Version 2.2.057 (Biostat Inc., Englewood, NJ, USA) (CMA; Borenstein, Hedges, Higgins, & Rothstein, 2005), Review Manager (RevMan) 5.3 (The Nordic Cochrane Center, The Cochrane Collaboration, Copenhagen, Denmark), and Minitab ^®^ statistical software version 19 (Minitab Inc., State College, PA, USA).

## 3. Results

### 3.1. Characteristics of the Included Studies

**Figure 1:**
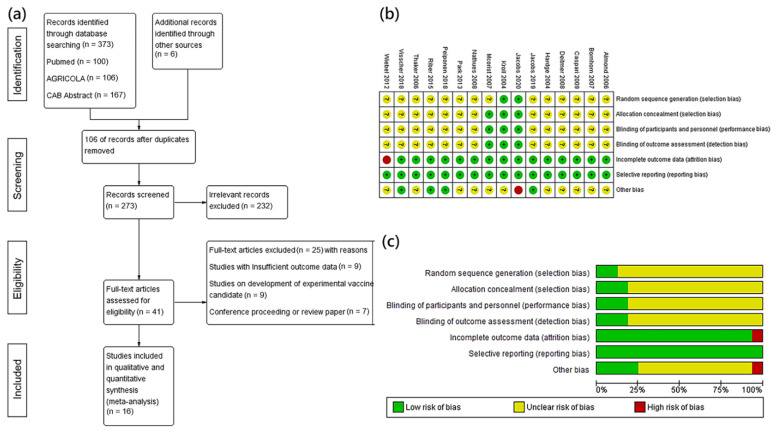
(**a**) Flowchart presenting article selection of the studies through the systematic review process and assessment of the risk of bias in this meta-analysis. (**b**) Summary of the risk of bias based on the Cochrane guideline. The symbols “+”, “−”, and “?” indicate low, high, and unclear risk of bias, respectively. (**c**) Risk of bias graph for each item expressed as percentages across the selected studies.

The processes for the search, screening, and selection of studies are summarized in Figure 1. The systematic database search identified 379 references; 41 were retained after the removal of duplicates and screening of titles and abstracts based on relevance (Figure 1a). Of these, 25 studies failed to meet one of the inclusion criteria (Table 1). Sixteen articles reporting 43 trials that evaluated commercial vaccines matched all inclusion criteria and reported at least one outcome of interest. The selected 16 studies were thoroughly reviewed to extract information for meta-analysis. A descriptive summary of each study included in the meta-analysis and the relevant outcome measures is shown in Table S1. The included studies were published between 2004 and 2020 in peer-reviewed journals. The majority of the studies were conducted in Europe, including Germany, Switzerland, Hungary, the Netherlands, and Demark. Two studies were from Australia, and one was published in South Korea. The summary showed that 13 trials were judged to have an unclear risk of bias, and three trials were considered to have a low risk of bias (Figure 1b,c). Since commercial vaccines were used and outcome assessments followed standard procedures, all studies seemed to have a low risk of bias for immunization performance and detection. Overall, a low-to-moderate risk of bias was observed in the studies included in the meta-analysis. 

### 3.2. Data Synthesis

#### 3.2.1. ADWG

**Figure 2:**
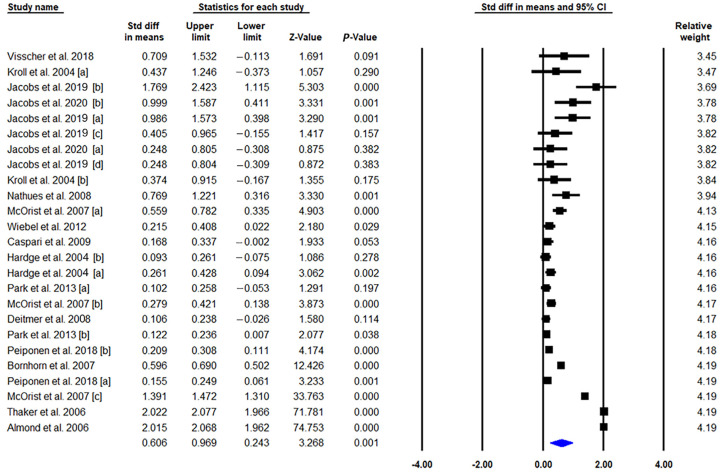
Meta-analysis forest plot of the effect of the commercial vaccines against *L. intracellularis* on ADWG for each of the trials using the pooled standardized mean difference (SMD). [a], [b], [c] and [d] indicated each trials containing interested outcomes in the selected studies. (Visscher et al., 2018 [43], Kroll et al., 2004 [a][b] [44], Jacobs et al., 2020 [a][b][c] [46], Jacobs et al., 2019 [a][b][c][d] [50], Nathues et al., 2008 [47], McOrist et al., 2007 [a][b][c] [48], Weibel et al., 2012 [7], Caspari et al., 2009 [6], Hardge et al., 2004 [a][b] [49], Park et al., 2013 [a][b] [38], Deitmer et al., 2008 [39], Peiponen et al., 2018 [a][b] [40], Bornhorn et al., 2007 [41], Thaker et al., 2006 [42], Almond et al., 2006 [3]).

Of the 15 studies that met the inclusion criteria [3,6,7,38,39,40,41,42,43,44,45,46,47,48,49], 25 trials that combined pigs immunized with live attenuated or inactivated vaccines (*n* = 16,147) and control groups (*n* = 15,487) were included in the pooled estimate. There was substantial heterogeneity among the trials (*I*^2^ = 99.449, *p* = 0.000); thus, a random-effects model was applied to the analysis. The meta-analysis results indicated that the immunized pigs had a greater ADWG than the control pigs (SMD = 0.606, 95% CI 0.243 to 0.969, *p* = 0.001, Figure 2). Sensitivity analysis, excluding trials with imputed SD, was performed to determine the robustness of the results.. For the sensitivity analysis with the trials not replacing missing data, the SMD was 0.723 (95% CI 1.208 to 0.238, z = 2.922, *p* = 0.003), indicating that SD imputation did not significantly influence the effect size, except that the range of variability decreased. Subgroup analysis was conducted to elucidate the potential source of inter-study heterogeneity with an a priori determined moderator: production phase, study type, and vaccine type. Based on these results (Table 2), a significant association between production phase and SMD was observed (*p* = 0.054). 

The largest SMD (0.720; 95% CI, 0.269–1.170) was observed in the subgroup with ADWG measured during the growing-finishing phase. The subgroup analysis based on the study type also showed a significant relationship between the field and controlled trials based on the effect size (*p* = 0.703). As with the subgroup analysis based on vaccine types, the difference was not calculated because of the limited number of available studies using the inactivated vaccine (*n* = 3). Taken together, the results of the meta-analysis demonstrated that commercial vaccination against LI infection had a significant influence on ADWG, one of the main production parameters. 

#### 3.2.2. Fecal Shedding Rate

**Figure 3:**
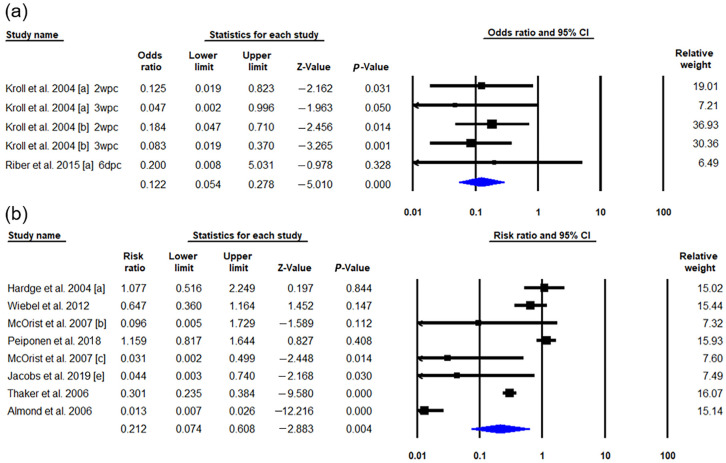
Meta-analysis forest plots comparing the effects of commercial vaccines against *L. intracellularis* using the pooled OR (**a**) or the pooled RR (**b**) with 95% CI. (**a**) Comparison of the reductions of fecal shedding between the vaccinated and control groups. (**b**) Mortality rate. [a], [b], [c] and [e] indicated each trials containing interested outcomes in the selected studies (Kroll et al., 2004 [a][b] [44], Riber et al., 2015 [a] [51], Hardge et al., 2004 [a] [49], Weibel et al., 2012 [7], Weibel et al., 2012 [7], McOrist et al., 2007 [b][c] [48], Peiponen et al., 2018 [40], Jacobs et al., 2019 [e] [50], Thaker et al., 2006 [42], Almond et al., 2006 [3]).

Six eligible publications reported fecal shedding as the outcome of interest. Of these, four studies were excluded from the meta-analysis due to the aggregation of outcome data [4,10,34] or unclear data for the time of vaccination or challenge infection [47]. Following the final screening process, two studies containing data from five trials were assessed to estimate the effect of vaccination on bacterial shedding [44,51], as determined by conventional PCR or quantitative PCR of fecal samples. The data were reported 6 days post-challenge (dpc) or 2 and 3 weeks post-challenge (wpc). Because no significant heterogeneity was observed among the effect sizes of each trial (Q = 1.073, *I*^2^ = 0%), a fixed-effects model was applied for analysis. The estimated odds ratio for shedding *L. intracellularis* in feces from vaccinated and control animals was 0.122 (95% CI 0.054–0.278, *p* = 0.008; Figure 3a). These pooled estimates indicated that vaccinated pigs were more likely to have reduced bacterial shedding following a challenge infection than control animals. With fecal shedding as the outcome, the meta-analysis was conducted using trials based on the period in which the animals were monitored after a challenge to eliminate potential heterogeneity bias. The analysis showed no statistical heterogeneity; therefore, no subgroup analysis was performed. 

#### 3.2.3. Mortality Rate 

For mortality rate as an outcome of interest, seven field studies comprising eight trials presented data for the meta-analyses [3,7,40,42,48,49,50]. Evidence of heterogeneity across the studies was identified; thus, a random-effects analysis was applied (*I*^2^ = 95.442, *p* = 0.000). The pooled estimated risk ratio (RR) of these eight trials was 0.199 (95% CI 0.066–0.605, *p* = 0.004; Figure 3b), indicating a significantly lower mortality risk in vaccinated animals (*n* = 12,392) compared to the controls (*n* = 13,562). Subgroup analysis was performed to reveal a potential source of inter-study heterogeneity with prespecified moderators: age of vaccination, production phase, and vaccine type. However, the production phase and vaccine type were excluded from the list of moderators because of the limited number of available trials. Of these eight trials, Peiponen et al., (2008) [40] only measured mortality risk during the nursery phase, and only Jacobs et al., (2019) [50] evaluated the effectiveness of the inactivated vaccine. In the subgroup analysis based on the age at vaccination, there was a significant association between the effect sizes (*p* = 0.076). The animals vaccinated at 10 weeks of age showed a lower risk ratio (0.058, 95% CI 0.004 to 0.759, *p* = 0.030) for mortality than those vaccinated at 3 weeks of age (0.552, 95% CI 0.224 to 1.362, *p* = 0.197). These results indicate that vaccination at an early age does not guarantee protection against PPE infection.

### 3.3. Publication Bias 

**Figure 4:**
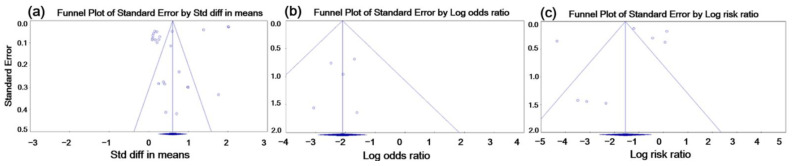
Funnel plots for testing publication bias for the selected trials included in the meta-analysis evaluating the effectiveness of the commercial vaccines against PPE for ADWG (**a**), fecal shedding rate (**b**), and mortality rate (**c**). The funnel plot shows the standard error of each trial on the vertical axis (precision) and the effect size of each study ((**a**), SMD; (**b**), pooled OR; (**c**), pooled RR) on the horizontal axis to investigate the magnitude of asymmetry showing publication bias.

Funnel plots representing the log risk ratio and standard error were used to assess potential publication bias (Figure 4). The funnel plot of the effect of vaccination on ADWG revealed a small degree of asymmetry (Figure 4), and Egger’s regression tests showed *p*-values of <0.05. The trim-and-fill test showed that the adjusted value (SMD = 0.706, 95% CI 0.393–1.019), compared with the observed value (SMD = 0.606, 95% CI 0.243–0.969), did not significantly change, indicating that publication bias was not a conclusive source of concern in this study. For fecal shedding or mortality rate, no asymmetry was observed in the funnel plot, and Egger’s test results indicated that there was no evidence of significant publication bias (*p* = 0.320 and *p* = 0.541, respectively).

## 4. Discussion

The present meta-analysis of 43 published trials investigating the effect of commercially available vaccines against *L. intracellularis* demonstrated the beneficial effect of vaccination on three different outcomes (i.e., effectiveness measures): ADWG, fecal shedding rate, and mortality rate, regardless of the type of vaccine. Regarding the vaccine types, too few trials of the inactivated vaccine were available to conduct a sub-group analysis, which resulted in the failure to clarify a statistically significant difference between vaccine types. One of the crucial advantages of vaccination is that it improves growth performance corresponding to increased ADWG (g/pig/day) in field conditions. The results of the meta-analysis showed that commercial vaccination against LI significantly increased the ADWG to 256 g and 720 g per day on average in the vaccinated pigs during the nursery and growing-finishing phases, respectively, compared to those of the controls (Table 2). This result indicated that the vaccination helps to protect the immunized pigs from the weight loss related to *L. intracellularis* infection, and the growth performance of the pigs also can be improved by the vaccination. A significant reduction in fecal shedding rate was also observed in vaccinated pigs. The odds of being positive for fecal bacterial DNA were reduced in the vaccinated group relative to those in the controls (0.122, 95% CI 0.054–0.278, *p* = 0.008) (Figure 4a). Only the trials conducted in an experimental setting with artificial challenges were included in the meta-analysis, as the challenge strain had to be identified to determine the PCR primers [44,51]. Given that *L. intracellularis* was not cultured in vitro [34], the high sensitivity of the PCR techniques may have resulted in the underestimation of the effectiveness of the vaccine in this pooled analysis [52]. To prevent a potential systematic bias associated with selecting the trials using the PCR method, the continuous scale of the PCR results was manually transformed to a dichotomous scale, and the pooled ORs were subsequently computed as the effect size. 

The pigs infected with LI, which show an acute clinical form of PPE, experience severe clinical implications, such as bloody manure, enterocyte necrosis, and high mortality, which may lead to significant economic losses ranging from 5.98 to 17.34 USD per marketed pig in the US [53]. Given the benefits of vaccination, such as economic productivity, reported by several studies relevant to veterinary vaccine development [54], VE is determined by three domains: study population, vaccination status, and mortality based on published literature, which was calculated as one minus the mortality risk ratio for vaccinated pigs and controls. In this meta-analysis, which included eight trials, the commercial vaccination against LI significantly reduced mortality by 80% (pooled RR: 0.199, CI 6.6–60.5, *p* = 0.004). These findings indicate that the beneficial effects of vaccination in preventing PPE are likely to be relatively high on swine farms. As the exact pathogenic mechanism of LI has not yet been elucidated [33], prevention seems to be the most effective intervention for at-risk pigs.

Because studies that reported the effectiveness of inactivated intramuscular vaccines and modified live oral vaccines [55] were included in the meta-analysis, the heterogeneity associated with the variation in vaccine protocols (i.e., dose, frequency, and routes) was minimized. To increase the accuracy of the pooled effect size in the meta-analysis, sampling errors caused by publication bias for positive findings were also examined. As shown in Figure 4, no significant publication bias related to negative results was observed. Despite these efforts to increase the internal validity of this study [56], all but three of the included studies were field trials conducted in a commercial setting, where the exposure rate was not homogeneous. VE could be increased in an experimental setting in which potential confounding factors could be controlled [57]. However, the in-field VE estimated from this meta-analysis can be easily generalized to the real-world impact of the vaccine (i.e., a high degree of external validity) [14], where stakeholders such as farmers have to decide whether to use commercial vaccines on their farms.

## 5. Conclusions

Considering that commercial vaccination is a less cost-effective strategy for controlling PPE compared with the prophylactic use of antibiotics [18], new vaccine candidate antigens have been identified, and alternative vaccine systems, such as live vector vaccines [26] or subunit vaccines with chimeric recombinant antigens [58], have been developed for use in practice. In this respect, the results of this meta-analysis and systematic review may provide industry stakeholders and researchers with updated information on the pooled effect of commercial vaccines and a summary of current scientific knowledge. Overall, the effectiveness of commercially available vaccines for protection against PPE-related weight loss, fecal shedding, and mortality was demonstrated in this meta-analysis and systematic review. Careful interpretation of the pooled efficacy estimates and the reported heterogeneity will be needed before the result is employed for evidence-based guidance during the decision-making process for vaccination recommendations for PPE control.

## Figures and Tables

**Table 1:** Primary reasons for the exclusion of 25 of 41 reviewed studies on swine vaccination against *L. intracellularis* from the meta-analysis.

Reason	References
Insufficient outcome data included	Riber et al., 2011a [14], Nogueira et al., 2013 [15], Bak et al., 2009 [16], Guedes et al., 2003 [17], Jansen et al., 2019 [18], Nogueira et al., 2015 [19], Riber et al., 2011b [20], Cordes et al., 2012 [21], Roerink et al., 2018 [4]
Studies involved with development of experimental vaccine candidates	Watson et al., 2011 [22], Watson et al., 2014 [23], Kim et al., 2017 [24], Park et al., 2019 [25], Park et al., 2018 [26], Won et al., 2018 [27], Obradovic et al., 2019 [28]
Conference proceeding or review paper	Rathkjen et al., 2007 [29], Gaumann et al., 2005 [30], Henke et al., 2006 [31], Obradovic et al., 2020 [32], Jacobson et al., 2010 [33], Kroll et al., 2005 [34], Okones et al., 2005 [35], Klien et al., 2011 [36], Dominique et al., 2014 [37].

**Table 2:** Subgroup analysis for the effect size (SMD) of ADWG; production phase and study types.

Sub-Group		No. of Trials	Point Estimate	Standard Error	Z-Value	*p*-Value	Heterogeneity (Total between)		
							Q-Value	Df (Q)	*p*-Value
Production phase	Nursery	10	0.256	0.005	3.580	0.000			
	Growing- finishing	15	0.720	0.230	3.131	0.002			
							3.711	1	0.054 *
Study type	Field trial	16	0.567	0.052	2.478	0.013			
	Controlled trial	9	0.675	0.027	4.074	0.000			
							0.145	1	0.703

Abbreviations: Df, degrees of freedom. * Values of *p* < 0.01 were considered statistically significant.

## Data Availability

All data used or analyzed during this study are included in this article and its Supplementary Materials.

## Supplementary Materials

The following supporting information can be downloaded at https://www.mdpi.com/article/10.3390/vetsci9100536/s1, Table S1: Characteristics and results of the eligible studies (trials) included in the systematic review and meta-analysis examining the effectiveness of commercial vaccines against *L. intracelluralis* in pigs.
