# Supplementary material for: The Effectiveness of Commercial Vaccination against Lawsonia intracellularis in Mitigating the Reduction in ADWG, the Increased Mortality and Fecal Shedding of the Vaccinated Pigs: A Systematic Review and Meta-Analysis
Source: Vet Sci. 2022 Sep 28;9(10):536. doi: 10.3390/vetsci9100536 (PMC9607650; doi:10.3390/vetsci9100536)
Supplement: Supplementary file 1 [file vetsci-09-00536-s001.zip › vetsci-1910971-supplementary.pdf]

Table S1. Characteristics and results of the eligible studies (trials) included in the systematic review and meta-analysis examining the effectiveness of commercial vaccines against *L. intracelluralis* in pigs.

Author (year)	Country	Setting	Vaccine type (dose, route)	Age of vaccination	Comparator	Challenge type	Production phase	Extracted outcome		
								ADWG (g/day); mean ± SD, sample size	Fecal shedding	Mortality (percentage)
Visscher et al. 2018 [43]	Germany	Controlled trial	Live (2ml, drench)	3w	Unvaccinated	Natural exposure	Nursery	V: 894 ± 73 (n = 9) C:821±114.3 (n = 18)	Excluded (the number of each group was not indicated)	NR
Kroll et al. 2004 [a] [44]	Australia	Controlled trial	Live (2ml, drench)	3w	unvaccinated	Artificial challenge (virulent LI isolate $10^{7.7}$TCID$_{50}$)	Nursery	V: 730 ± 160.9 (n = 15) C: 660 ± 159.5 (n = 10)	2wpc; V: 5/15, C:8/10 3wpc; V:0/15, C:4/10	NR
Kroll et al. 2004 [b] [44]			Live (2ml, drench)[c]					V: 550 ± 160.9 (n = 40) C: 490 ± 159.5 (n = 20)	2wpc; V: 6/20, C:14/20 3wpc; V:4/20, C:15/20	
Peiponen et al. 2018 [a] [40]	Finland	Field trial	Live (2ml, drench)	3w	Placebo with sham vaccination (Circo virus)	Natural exposure	Nursery	V:325.1 ± 123.1 (n = 842) C: 306 ± 123.3 (n = 905)	NR	V: 6.5% (64/985)[a] C: 5.6% (55/981)
Peiponen et al. 2018 [b] [40]							Growing-finishing	V: 938.6 ± 154 (n = 800) C: 907.7 ± 141 (n = 800)		
Jacobs et al. 2020 [a] [46]	Netherland	Controlled trial	Inactivated (0.2ml. ID)	3w	unvaccinated	Artificial challenge (Intestinal	Nursery	V: 956 ± 119 (n = 25)	Excluded (only continuo	NR

						mucosa homogenate from infected pig)		C: 674 ± 381 (n = 25)	us data provided)	
Jacobs et al. 2020 [b] [46]			Live (2ml, drench)				Nursery	V: 812 ± 287 (n = 25) C: 674 ± 381 (n = 25)		
Nathues et al. 2008 [47]	Germany	Field trial	Live (2ml. drinking or drench)	11w or 8-9w	unvaccinated	Natural exposure	Growing-finishing	V: 669.1 ± 35[b] (n = 60) C:644.5 ± 24.8[b] (n = 30)	Excluded : timeline following the challenge was not indicated	NR
McOrist et al. 2007 [a] [48]	Australia	Field trial	Live (2ml. drinking)	3-6w	Placebo with zinc oxide	Natural exposure	Growing-finishing	V: 830 ± 160.9 (n = 160) C: 770 ± 108.7 (n = 160)	NR	Excluded: no death was caused by PPE
McOrist et al. 2007 [b] [48]								V: 790 ± 160.9 (n = 378) C: 760 ± 108.7 (n = 399)		V: 0% (0/378)[h] C: 1.2% (5/399)
McOrist et al. 2007 [c] [48]								V: 850 ± 160.9 (n = 1076) C: 700 ± 159.5 (n = 2139)		V: 0% (0/1076) C: 1.5%[c] (32/2139)
Weibel et al. 2012 [7]	Switzerland	Field trial	Live (2ml, drench)	3w	Placebo with adjuvant	Natural exposure	Growing-finishing	V: 774 ± 106 (n = 292) C: 751 ± 108.7 (n = 160)	NR	V: 5.3% (17/318) C: 8.3% (27/327)
Caspari et al. 2009 [6]	Switzerland	Field trial	Live (2ml, drench)	3w	Placebo with 0.9%NaCl	Natural exposure	Growing-finishing	V: 795 ± 106 (n = 256) C: 777 ± 108.7 (n = 279)	NR	NR
Hardge et al. 2004 [a] [50]	Germany	Field trial	Live (2ml, drench)	7w	Unvaccinated	Natural exposure	Growing-finishing	V: 702 ± 106 (n = 278) C: 674 ± 108.7 (n = 278)	NR	V: 4.98% (14/278) C: 4.68% (13/278)

Hardge et al. 2004 [b] [50]				3w				V: 692 ± 106 (n = 273) C: 682 ± 108.7 (n = 271)		Excluded (mortality partially caused by respiratory syndrome)
Park et al. 2013 [a] [38]	Korea	Field trial	Live (2ml, PO)	32 days	Placebo with sham vaccinations (PRRS, swine erysipelas, Japanese encephalitis, hog cholera vaccine)	Natural exposure	Nursery	V: 509 ± 86 (n = 323) C: 500 ± 90 (n = 315)	NR	Excluded (total death number was not provided)
Park et al. 2013 [b] [38]							Growing-finishing	V:736.8 ± 155.3 (n = 595) C:716.8 ± 173.5 (n = 575)		
Deitmer et al. 2008[39]	Germany	Field trial	Live (2ml, PO)	32 days	Placebo with Tylosin	Natural exposure	Nursery	V: 421 ± 160.9 (n = 470) C: 404 ± 159.5 (n = 421)	NR	NR
Bornhorn et al. 2007 [41]	Germany	Field trial	Live (2ml, PO)	3w	Placebo with Tylosin	Natural exposure	Growing-finishing	V: 833 ± 41.7[d] (n = 2208) C: 808 ± 42.7[d] (n = 560)	NR	NR
Thaker et al. 2006 [42]	Hungary	Field trial	Live (2ml, drench)	10w	Unvaccinated	Natural exposure	Growing-finishing	V: 799 ± 49 (n = 3810) C: 650 ± 92 (n = 3809)	NR	V: 2% (80/3810) C: 6.9% (266/3809)
Almond et al. 2006 [3]	Hungary	Field trial	Live (2ml, drinking)	9-11w	Unvaccinated	Natural exposure	Growing - Finishing	V: 780 ± 45 (n = 4112) C: 660 ± 71 (n = 4188)	NR	V: 0.2% (8/4112) C: 14.9% (624/4188)
Jacobs et al. 2019 [a] [49]	Netherland	Controlled trial	Inactivated (2ml. IM)	4w	Unvaccinated	Artificial challenge (Intestinal mucosa homogenate from infected pig)	Nursery	V: 935 ± 306 (n = 25) C: 550 ± 460 (n = 25)	Excluded (continuous data only provided)	NR
Jacobs et al. 2019 [b] [49]							Growing - Finishing	V: 956 ± 119 (n = 25)		

								C: 674 ± 381 (n = 25)		
Jacobs et al. 2019 [c] [49]			Live (2ml, drench)				Nursery	V: -229 ± 1301 (n = 25) C: -655 ± 723 (n = 25)		
Jacobs et al. 2019 [d] [49]							Growing - Finishing	V: 655 ± 385 (n = 25) C: 550 ± 460 (n = 25)		
Jacobs et al. 2019 [e] [49]		Field trial	Inactivated	12w	Placebo with sham-vaccination (PRRS vaccine)	Natural exposure	Growing - Finishing	Excluded (control group came from historical data)	NR	V: 0% (0/1435) C: 0.7% (11/1441)
Riber et al. 2015[51]	Denmark	Controlled trial	Live (2ml, drench)	5-6w	Placebo with antibiotics (tiamulin)	Artificial challenge (10^{10} virulent LI strain)	NR	Excluded (only qualitative analysis provided)	6dpc; V: 6/8, C:6/7 14dpc; V:8/8, C:7/7 18dpc; [e] V: 5/5, C:7/7	NR

Abbreviations: NR, not reported; wpc, week post-challenge; PO, per oral; CI, confidence interval

[a] The number of death estimated based on the percentage and case number

[b] Data extracted from the graph to include both mean and SD value

[c] Mortality rate caused by PPE in the control group was estimated and ranged between 1.5%-11.3%. For data analysis, a 1.5% mortality rate was selected.

[d] The pooled data were obtained from eight farms.

[e] The number of samples is not consistent in the vaccinated group, since some data were missing in the original study.

References

3. Almond, P.K.; Bilkei, G. Effects of Oral Vaccination against *Lawsonia Intracellularis* on Growing-Finishing Pig's Performance in a Pig Production Unit with Endemic Porcine Proliferative Enteropathy (PPE). Dtsch. Tierarztl. Wochenschr. 2006, 113, 232–235

6.Caspari, K.; Kummerlen, D.; Voets, H.; Eichin, E.; Zeeh, H.; Zimmermann, W. Field study about the use of Enterisol Ileitis in a swine herd in Switzerland. Schweiz. Arch.

Tierheilkd. 2009, 151, 31–32. https://doi.org/10.1024/0036-7281.151.1.31
7.Weibel, H.; Sydler, T.; Brugnera, E.; Voets, H.; Grosse Liesner, B.; Sidler, X. Efficacy of Simultaneous Vaccination with En-terisol(R) Ileitis and Ingelvac(R) CircoFLEXTM in a Swiss Breeding Farm. Schweiz. Arch. Tierheilkd. 2012, 154, 445–450. https://doi.org/10.1024/0036-7281/a000381.
38.Park, S.; Lee, J.-B.; Kim, K.-J.; Oh, Y.-S.; Kim, M.-O.; Oh, Y.-R.; Hwang, M.-A.; Lee, J.-A.; Lee, S.-W. Efficacy of a Commercial Live Attenuated *Lawsonia Intracellularis* Vaccine in a Large Scale Field Trial in Korea. Clin. Exp. Vaccine Res. 2013, 2, 135–139. https://doi.org/10.7774/cevr.2013.2.2.135.
39.Deitmer, R.; Bubikat, A.; Keller, C.; Adam, M.; Voets, H. Embedded Ileitis Vaccination or Antibiotic Treatment-a Compari-son of Efficacy and Economic Impact during the Nursery Period. Prakt. Tierarzt-Hann. 2008, 1, 142–151.
40.Peiponen, K.S.; Tirkkonen, B.T.; Junnila, J.J.T.; Heinonen, M.L. Effect of a Live Attenuated Vaccine against *Lawsonia Intracellularis* in Weaned and Finishing Pig Settings in Finland. Acta Vet. Scand. 2018, 60, 18. https://doi.org/10.1186/s13028-018-0374-8.
41.Bornhorn, R. Efficacy and Economical Impact of Oral Vaccination of Partially Infected Piglets with Enterisol (R) Ileitis. Prakt. Tierarzt 2007, 88, 172–178.
42.Thaker, M.Y.C.; Bilkei, G. Comparison of the Effects of Oral Vaccination and Different Dietary Antibiotic Prophylactic Treatment against *Lawsonia Intracellularis* Associated Losses in a Fattening Pig Production Unit with High Prevalence of Porcine Proliferative Enteropathy (PPE). Tierarztl. Umsch. 2006, 61, 372–376.
43.Visscher, C.; Kruse, A.; Sander, S.; Keller, C.; Mischok, J.; Tabeling, R.; Henne, H.; Deitmer, R.; Kamphues, J. Experimental Studies on Effects of Diet on *Lawsonia Intracellularis* Infections in Fattening Boars in a Natural Infection Model. Acta Vet. Scand. 2018, 60, 22. https://doi.org/10.1186/s13028-018-0378-4.
44.Kroll, J.J.; Roof, M.B.; McOrist, S. Evaluation of Protective Immunity in Pigs Following Oral Administration of an Avirulent Live Vaccine of *Lawsonia Intracellularis*. Am. J. Vet. Res. 2004, 65, 559–565. https://doi.org/10.2460/ajvr.2004.65.559.
46.Jacobs, A.A.C.; Harks, F.; Pauwels, R.; Cao, Q.; Holtslag, H.; Pel, S.; Segers, R. Efficacy of a Novel Intradermal *Lawsonia Intracellularis* Vaccine in Pigs against Experimental Infection and under Field Conditions. Porc. Health Manag. 2020, 6, 25. https://doi.org/10.1186/s40813-020-00164-0.
47.Nathues, H.; Grosse Beilage, E. Diagnosis of *Lawsonia Intracellularis* Infection in Pigs after Vaccination or Antimicrobial Treatment. Dtsch. Tierarztl. Wochenschr. 2008, 115, 404–409.
48.McOrist, S.; Smits, R.J. Field Evaluation of an Oral Attenuated *Lawsonia Intracellularis* Vaccine for Porcine Proliferative En-teropathy (Ileitis). Vet. Rec. 2007, 161, 26–28. https://doi.org/10.1136/vr.161.1.26.
49. Jacobs, A.A.C.; Harks, F.; Hazenberg, L.; Hoeijmakers, M.J.H.; Nell, T.; Pel, S.; Segers, R.P.A.M. Efficacy of a Novel Inactivated *Lawsonia Intracellularis* Vaccine in Pigs against Experimental Infection and under Field Conditions. Vaccine 2019, 37, 2149–2157. https://doi.org/10.1016/j.vaccine.2019.02.067.Hardge, T.; Nickoll, E.;
50. Grunert, H.; Elbers, K.; Langbein, U.; Keller, C.; Bleier, T.; Pohlenz, J.; Ohlinger, V.F.; Schroeder, B. Prevention of Porcine Proliferative Enteropathy (PPE) by Vaccination—Efficacy and Economics in European Farms. Pig J. 2004, 54, 17–34.
51.Riber, U.; Heegaard, P.M.H.; Cordes, H.; Ståhl, M.; Jensen, T.K.; Jungersen, G. Vaccination of Pigs with Attenuated *Lawsonia Intracellularis* Induced Acute Phase Protein Responses and Primed Cell-Mediated Immunity without Reduction in Bacterial Shedding after Challenge. Vaccine 2015, 33, 156–162. https://doi.org/10.1016/j.vaccine.2014.10.084.
